# Identification of bZIP transcription factors and their responses to brown spot in pear

**DOI:** 10.1590/1678-4685-GMB-2021-0175

**Published:** 2022-01-31

**Authors:** Li Liu, Yuxin Zhang, Qi Wang, Xingyu Tao, Jing Fang, Wenjuan Zheng, Liwu Zhu, Bing Jia, Wei Heng, Shaowen Li

**Affiliations:** 1 Anhui Agricultural University, School of Horticulture, Hefei, Anhui, P.R. China.; 2 Anhui Agriculture University, School of Information and Computer Science, Hefei, Anhui, P. R. China.

**Keywords:** Pear, *bZIP* gene family, brown spot, hormone, expression profile

## Abstract

Basic leucine zipper (bZIP) is a conserved transcription factor (TF) widely present in eukaryotes, and it plays an important role in regulating plant growth and stress responses. To better understand the white pear *bZIP* gene family, comprehensive bioinformatics analysis of the pear genome was performed. A total of 84 *PbbZIP* genes were identified, which were divided into 13 subfamilies by phylogenetic analysis. The 84 *PbbZIP* genes were all located in the nucleus, and 77 of those genes were unevenly distributed across the 17 chromosomes of white pear. The other 7 *PbbZIP* genes were located on the scaffold. Subsequent expression profile analysis showed that *PbbZIP* genes in exocarp were significantly upregulated or downregulated in ‘Huangguan’ pear with brown spot (BS) compared with healthy pear and in response to hormonal treatment with gibberellin A_3_ (GA_3_). These results provide helpful insights into the characteristics of *PbbZIP* genes and their responses to BS in ‘Huangguan’ pear.

## Introduction

Basic leucine zippers (bZIPs) play important regulatory roles in many key biological processes and are each comprised of a DNA binding domain and a leucine zipper dimer domain; they are one of the largest gene families and the most widely distributed class of eukaryotic TFs (Glover et al., 1995; Amorim et al., 2017). bZIP family members each have a highly conserved domain comprised of 60~80 amino acids, and they can be divided into 10 subfamilies that exhibit different expression levels and perform different functions in different plants and tissues. bZIP transcription factors (TFs) have been identified in many plants, such as *Arabidopsis* (Jakoby et al., 2002), Gramineae (Wei et al., 2012; Nijhawan et al., 2008; Wang et al., 2019), olive (Rong et al., 2020), and many horticultural crops (Wang *et al*., 2017; An et al., 2018; Lu et al., 2020; Jin et al., 2021).

During plant growth and development, bZIP TFs play important roles in seed maturation and germination, cell elongation, flower induction and development, hormone stress and so on (Wang et al., 2011). bZIP TFs are also involved in plant responses to biotic and abiotic stresses. For example, the expression of bZIP related genes can be induced under drought, salt, cold stress, and auxin (IAA) and abscisic acid (ABA) hormone treatment (Hossain et al., 2010). In cucumber, *CsbZIP12* and *CsbZIP44* genes have been found to be upregulated in the roots after drought stress treatment (Baloglu et al., 2014). In tomato, the bZIP TF SIAREB is involved in the response to water deficiency and salt stress (Hsieh *et al*., 2010). In ‘Gala’ apple, the expression of *MdAREB2* has been shown to increase rapidly after ABA treatment and thereby affect the expression of some stress-resistant genes under high temperature or light stress (Liu et al., 2018). In addition, the expression of *MdbZIP26* was found to be significantly upregulated under drought and salt stresses, or exogenous ABA treatment thereby enhancing plant stress resistance through the ABA signaling pathway. This evidence suggests that the bZIP family is involved in plant stress resistance through hormone signaling.

White pear (*Pyrus bretschneideri* Rehd.) is a deciduous fruit tree of *Pyrus* genus of Rosaceae (Teng et al., 2004), and its fruit is juicy, sweet and refreshing and is well favored by consumers. Complete genome sequencing of pear has laid a foundation for biological information analysis of the white pear TF family. Many TF families have been characterized, such as WRKY and NAC (Huang et al., 2015; Gong et al., 2019), but there have been no reports on the bZIP TF family in white pear. 

In this study, a bioinformatics method was used for genome-wide identification of the *bZIP* gene family in white pear, and the gene structure, physicochemical properties, conserved motifs, phylogenetic relationships, chromosomal locations, collinearity and *cis*-acting elements of *bZIP* family members were analyzed. In our previous study, we found that gibberellin A_3_ (GA_3_) has a promoting effect on brown spot (BS) (Wang et al., 2021). In the present study, the expression levels of *bZIP* genes in exocarp of ‘Huangguan’ pear were evaluated in normal fruit, fruit with BS and fruit treated with GA_3_. These results enhance our understanding the characteristics of the *bZIP* gene family in the pear genome and provide insights into how bZIPs participate in regulating BS on the skin of ‘Huangguan’ pear through gibberellin (GA) signaling.

## Material and Methods

### Plant material

‘Huangguan’ pear was used as a material in this study, and its fruit samples were grown in a 30-year-old horticulture orchard (Dangshan, Anhui, China). GA_3_ (300 mg/L, Sigma G8040) and water were sprayed on ‘Huangguan’ pear at 10, 20 and 30 days after full bloom (DAFB). After treatment, the fruits were bagged individually. Each treatment had three biological replicates, and each tree had approximately 120 treated fruits. All fruit samples were collected during the harvest season in 2018. The peel was cut into slices manually, immediately frozen in liquid nitrogen, and stored at -80 °C. 

**Identification of *PbbZIP* family members**

The PF00170 and PF07716 domain model files of the bZIP family members were downloaded from the PFAM website (https://www.pfam.org), and the coding sequence (CDS) nucleotide sequence files, transcript amino acid sequence files and gene annotation files were downloaded from the Genome Database for Rosaceae (GDR) (https://www.rosaceae.org) and the Phytozome Genome Data Resource Library (https://genome.jgi.doe.gov/portal). The first batch of candidate genes containing PF00170 and PF07716 domains was identified (E-value=e^-10^) using HMMER v.3.2 (Yap et al., 2016). 

The *AtbZIP* genes were obtained by querying the *Arabidopsis* database (TAIR) (https://www.arabidopsis.org/), and a local BLAST library was constructed using the amino acid sequence files of each species. Then, a BLASTP search was carried out using the local BLAST library (E-value= e^-10^) with the AtbZIP amino acids, and another batch of candidate genes was obtained. 

The two batches of candidate genes were merged and the domains were identified using HMMSCAN (Finn et al., 2015) (E-value=e^-10^). Low similarity and repetitive sequences were manually removed. Finally, the prephenate dehydratase and ACT domains of the family members were identified using SMART (http://smart.embl- heidelberg.de) and PFAM (https://www.pfam.org), respectively. 

**Phylogenetic analysis of the *PbbZIP* family genes**

The bZIP protein sequences of white pear and *Arabidopsis* were extracted and aligned. The InterProScan program was used on all of the candidate protein pairs and confirmed the presence of the diagnostic domain using the Pfam and SMART databases. MAFFT was used with the default parameters to align the sequences of the multiple homologous bZIP genes. A phylogenetic tree was constructed using the maximum likelihood method and IQ-TREE 1.6.9 software (Nguyen et al., 2015). The support values displayed next to the branches were inferred from 1000 replicate trees. 

**Gene structural and conserved motif analyses of the *PbbZIP* family genes**

Based on the amino acid sequences, the conserved motifs of the *PbbZIP* family members were analyzed with Motif EM for Motif Elicitation (MEME) v.5.0 software (Bailey et al., 2009). The motif value was set to 10, and the minimum and maximum motif lengths were set to 6 and 50, respectively.

The chromosome position information of the *bZIP* family members was extracted from the gene annotation files. The major features, including the coding and noncoding regions and the exon-intron pattern, were characterized with Gene Structure Display server (GSDS) v.2.0 software (http://gsds.cbi.pku.edu.cn/index.php). The amino acid sequences of the bZIPs in pear and *Arabidopsis* were aligned and divided into different domains using Jalview v.2.10 software (Waterhouse et al., 2009).

**Analysis of the physical and chemical properties of the *PbbZIP* familiy genes**

The protein physicochemical properties of the bZIP family members were predicted via the ExPASy website (https://www.expasy.org/) (Gasteiger et al., 2003). The signal peptides were analyzed through SignalP (http://www.cbs.dtu.dk/services/SignalP/) (Armenteros et al., 2019). The subcellular locations were calculated using CELLO v.2.5 software (http://cello.life.nctu.edu.tw/) (Yu et al., 2004). The transmembrane structures were predicted by TMHMM Server v.2.0 software (http://www.cbs.dtu.dk/services/TMHMM/) (Krogh et al., 2001).

**Gene collinearity relationships of the *PbbZIP* family genes**

The multiple collinearity scan toolkit (MCScanX) (Wang et al., 2012) was used for analysis of collinearity between multiple genomes and the pear genome, and the homologous regions between pears and *Arabidopsis* were anchored. Gene collinearity relationships were visualized using the Python package circos (https://github.com/Tanghaibao/circos).

**Transcriptional profiling of the *PbbZIP* family genes**

An RNA-seq dataset (PRJNA682706) of pear subjected to several different treatments, including NaH_2_PO_4_·2H_2_O (0.2%, Sigma 04269), ABA (100 μM, Sigma A1049), and GA_3_ (300 mg/L, Sigma G8040), as well as normal fruits and fruits with BS disease, was obtained from our previous work (Wang et al., 2021). Each had three biological replicates. Data from normal fruit, fruit with BS and fruit treated with GA_3_ was used in this study. The raw data were filtered, and the fragments per kilobase of transcript per million mapped reads (FPKM) values were calculated and investigated for the expression of *PbbZIP* members. A heatmap of the gene expression profiles of all *PbbZIP* genes was constructed using TBtools software (Chen et al., 2020). 

**Coexpression network of *PbbZIP* genes and GA signaling genes in BS formation**

To explore the expression patterns of *PbbZIP* genes and GA signaling genes during BS formation, RNA sequencing (RNA-seq) data of exocarp of healthy ‘Huangguan’ pear and ‘Huangguan’ pear with BS disease were used to measure the expression similarity between gene pairs as Pearson’s correlation coefficient (PCC) values. The values were then filtered with Excel software (with the parameter set as > 0.4). Visualizations of the data were performed with Cytoscape software (Shannon et al., 2003).

**qRT-PCR analysis of *PbbZIP* genes**

To confirm the expression of the *PbbZIP* genes, total RNA was extracted from each fruit sample with a total RNA purification kit and used to perform reverse transcription with the Prime Script™ RT Reagent Kit. Beacon Designer 7.9 software was used to design the specific primers (Table S1). qRT-PCR was conducted in a 20 µL reaction volume that comprised 10 µL SYBR Premix ExTaq II, 2 µL template cDNA, 0.8 µL each of forward and reverse primer and the remaining volume with nuclease-free water. The PCR conditions were as follows: 94°C for 45 s, followed by 40 cycles of 94°C for 15 s, 60°C for 20 s and 72°C for 20 s; and then holding at 4°C forever. GAPDH in pear was used as the reference gene. The 2^-ΔΔCT^ method was used to calculate the relative expression levels of the *PbbZIP* genes.

## Results

**Identification of bZIP TFs in *P. bretschneideri***

Genome-wide analysis was performed to search for *bZIP* members in the *P. bretschneideri* genome (Wu et al., 2013). Eighty-four *bZIP* genes were identified in pear by Pfam and inter-ProScan confirmation, and several manual checks were performed. Based on their positions on the chromosomes, the *bZIP* genes in pear were denominated as *PbBZIP1*-*PbBZIP84* (Table S1). Our analysis confirmed that all of the identified *PbbZIP* proteins each contained conserved bZIP_1 (PF00170) and bZIP_2 (PF07716) domains, which are the specific conserved domains of the *PbbZIP* gene family.

The lengths of the *PbbZIP* genes ranged from 369 bp to 2,229 bp, and the average length was 1,021 bp. The *PbbZIP* proteins contained 123 to 743 amino acids, with an average of 340 amino acids. Molecular weight (MW) ranged from 30,140.67 Da to 183,693.94 Da, with an average of 83,865.54 Da. The predicted isoelectric points of the *PbbZIP* proteins ranged from 4.91 to 5.23 (Table S2). In addition, all 84 *PbbZIP* genes were predicted to be located in the nucleus.

**Phylogenetic relationships of the *PbbZIP* proteins**

**Figure 1 -:**
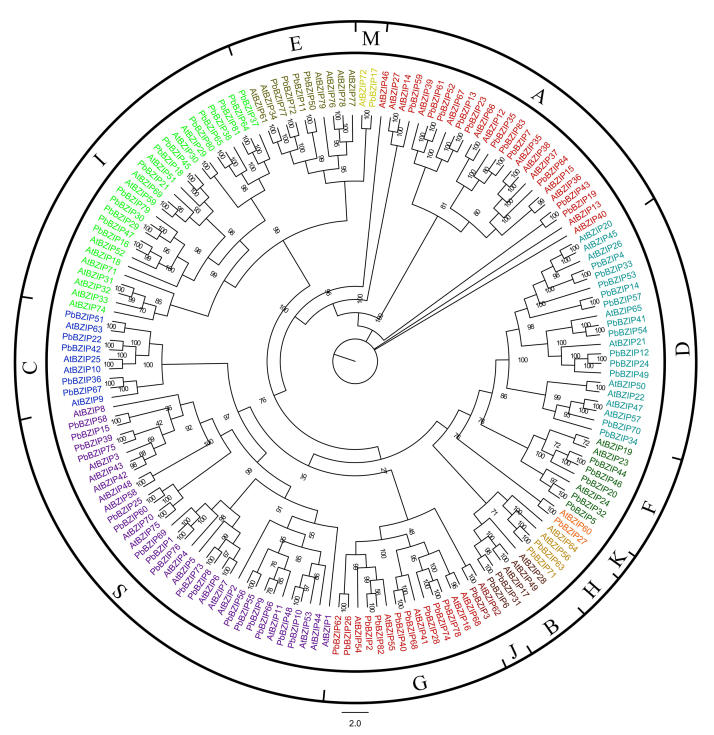
Phylogenetic analysis of *bZIPs* from pear and *Arabidopsis thaliana*. The tree was constructed with IQ-TREE v.1.6 software. The bZIP TFs clustered into 13 distinct clades, marked by curves of different colors.

To further investigate the phylogenetic relationships among the bZIP proteins in *P. bretschneideri* and *Arabidopsis*, a phylogenetic tree was constructed. The tree showed that the bZIP proteins of Chinese white pear and *Arabidopsis* could be divided into 13 subfamilies (denoted Groups A, B, C, D, E, F, G, H, I, J, K, S and M). *PbbZIP* genes in both *P. bretschneideri* and *Arabidopsis* contributed to all of the subfamilies A-M (Figure 1). Among these subfamilies, Group S had the largest number of *PbbZIP* gene members, 17, while Groups J, K and M had the smallest number, 1. These results may indicate special functions of bZIP members in white pear.

**Conserved structure and intron-exons of the *PbbZIP* gene family**

**Figure 2 -:**
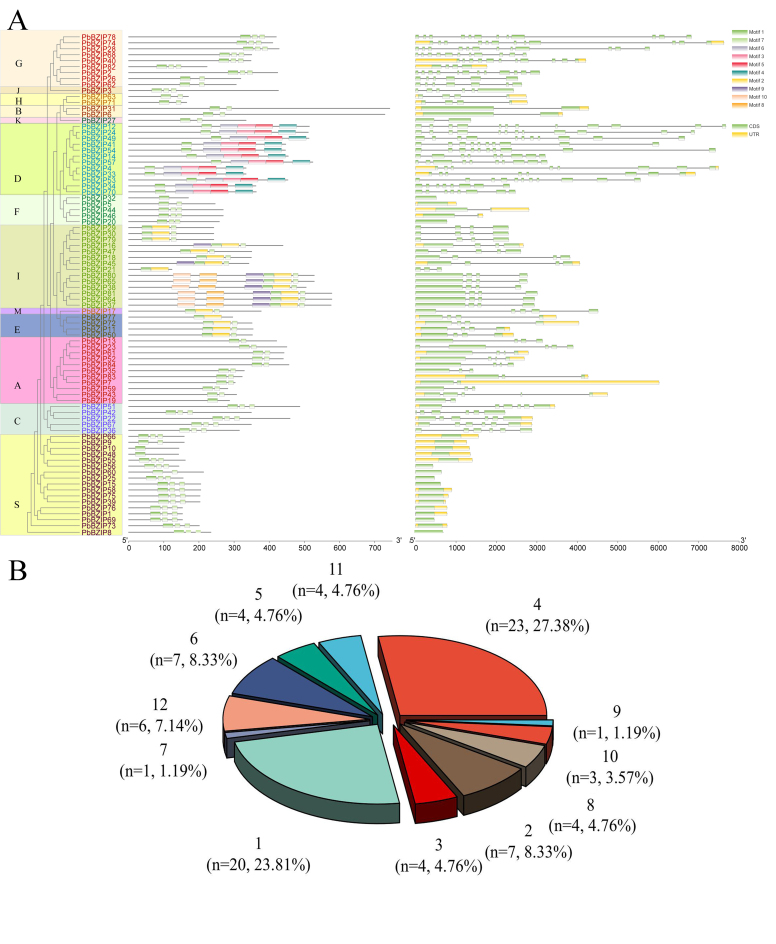
Phylogenetic relationships, motif compositions and gene structures of *PbbZIPs*. (A) The phylogenetic tree was produced by MEGA using the neighbor-joining method with 1,000 bootstrap replicates. (B) A schematic representing the conserved motifs of the *PbbZIPs* identified by MEME. Each motif is indicated by a colored box numbered at the bottom. (C) Exon number distribution of the *PbbZIP* family TFs in white pear.

The genomic structures of the 84 *PbbZIP* genes were quite different from one another. All contained at least one exon, and the maximal number was 12 (Figure 2B, Table S3). Six *PbbZIPs* contained 12 exons (7.14%), 4 members contained 11 exons (4.76%), 3 contained 10 (3.57%), 1 contained 9 (1.19%), 4 contained 8 (4.76%), 1 contained 7 (1.19%), 7 contained 6 (8.33%), 4 contained 5 (4.76%), 23 contained 4 (27.38%), 4 contained 3 (4.76%), 7 contained 2 (8.33%) and 20 contained 1 (23.81%). In addition, the members within a subfamily had similar gene structures, including similar lengths and numbers of exons, which supported the classification and the identified evolutionary relationships.

In total, 10 motifs were detected from the *PbbZIP* genes. Within a subfamily, the numbers and types of conserved motifs of *PbbZIP* were similar, while these were quite diverse among the different subfamilies (Figure 2A). Motif 1 domain is present in all *PbbZIP* proteins. In addition, some motifs have obvious specificity and belong to specific subgroups. For example, motif 6, motif 3, motif 5, and motif 4 only appear in the D subfamily; motif 8, motif 9 and motif 10 only appear in the I subfamily; and motif 2 only appears in the I and M subgroups. The other subgroups have only the motif 1 domain. Different subgroups with specific motifs may contribute to specific functions.

**Chromosomal locations and syntenic relationships of the *PbbZIP* genes**

**Figure 3 -:**
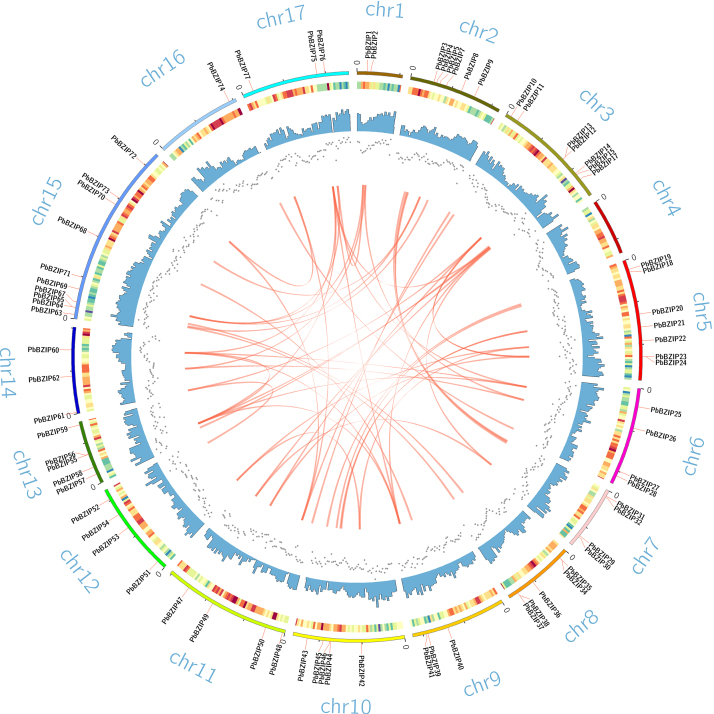
The chromosomal locations of 77 *PbbZIPs*. The genes were mapped to the pear chromosomes by TBtools. The chromosomes of pear are shown arranged in a circle. The outer circle represents the chromosome. Heatmaps, scatterplots and histograms were used to record the gene density of each chromosome segment. The length of each chromosome segment was set at 500,000 bp.

To determine the distribution of the *PbbZIP* genes on the pear chromosomes, their chromosomal locations were visualized with MCScanX software. The 77 *PbbZIP* genes were found to be randomly and unevenly distributed on the 17 chromosomes across the white pear genome and the other 7 *PbbZIP* genes were located on the scaffold (Figure 3, Table S4). The number of *PbbZIP* genes on each individual chromosome ranged from 0 (Chr4) to 11 (Chr15). The same number of *PbbZIP* genes (5) were distributed on chromosomes 7, 8, 10 and 13. There were eight *PbbZIP* genes located on chromosomes 3 and 5. Chromosomes 6, 11 and 12 harbored four *PbbZIP* genes, and three *PbbZIP* genes were located on chromosomes 9, 14 and 17. 

Gene tandem and fragment repeats are key factors that promote the number of genes in a particular gene family and lead to functional diversity during evolution. In this study, three pairs of *PbbZIP* genes (*PbbZIP34/PbbZIP35, PbbZIP37/PbbZIP38, PbbZIP64/PbbZIP65*) were identified as the result of tandem duplication, and 53 pairs were identified as the result of segmental duplication, indicating that segmental duplication was the main force driving the expansion of the *PbbZIP* family in pear. 

**Phenotype of ‘Huangguan’ pear and the expression patterns of the *PbbZIP* genes**

**Figure 4 -:**
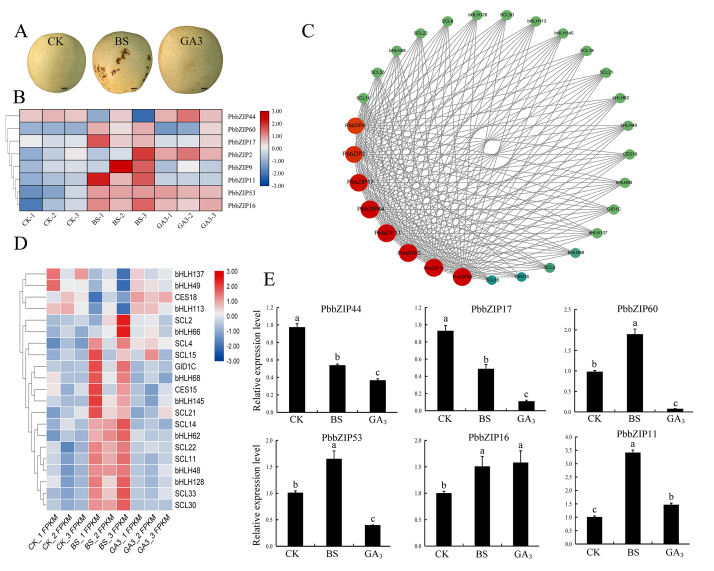
Phenotypic characteristics of BS, expression profiles of *PbbZIP* genes and a coexpression network and heatmap for *PbbZIP*s with GA signaling genes in pear. (A) Phenotypes of normal ‘Huangguan’ pear (CK), ‘Huangguan’ pear with BS disease and ‘Huangguan’ pear treated with GA_3_. (B) The expression patterns of *PbbZIP* genes in ‘Huangguan’ pear. (C) Interaction network analysis of *PbbZIPs* with GA signaling genes in pear was performed using the STRING database. (D) Heatmap of GA signaling genes expressed in ‘Huangguan’ and ‘Huangguan’ with BS based on the fold change (log2) in FPKM values. The color scale at the top represents log2-FPKM values. (E) Relative expression profiles of *PbbZIP* genes in the exocarp of ‘Huangguan’ pear. Error bars represent the SE of three biological replicates. Lowercase letters indicate significant differences between treatments at the *P* < 0.05 level. CK: normal ‘Huangguan’ pear; BS: ‘Huangguan’ pear with BS; GA_3_: ‘Huangguan’ pear treated with GA_3_.

The phenotypes of healthy ‘Huangguan’ pear, ‘Huangguan’ pear with BS and ‘Huangguan’ pear treated with GA_3_ were observed. The results showed that the fruit size increased significantly after GA_3_ treatment (Figure 4A). In our previous study, we found that the GA signaling pathway may be the key pathway regulating the occurrence of BS (Wang et al., 2021). Based on the transcriptome analysis, 8 *PbbZIP* genes were identified, most of which were significantly upregulated in pears with BS compared with healthy pears. Among them, *PbbZIP44*, *PbbZIP2*, *PbbZIP53* and *PbbZIP16* were also significantly upregulated with GA_3_ treatment (Figure 4B, Table S4). These results suggest that the expression of some *PbbZIP* genes can be activated by GA_3_ treatment and promote the occurrence of BS, which demonstrates that *PbbZIP* family genes play an important role in regulating the formation of BS and are responsive to GA_3_ hormone.

### Coexpression network of BS-related genes 

Coexpression network analysis was performed to illuminate the collaboration among the *PbbZIP* genes, analysis of the transcriptome data showed that 8 *bZIP* genes could be classified into different coexpression clusters with GA signaling genes (Figure 4C, Table S5). These *PbbZIP* genes had high correlations with 21 GA signaling genes, which were highly expressed in BS (Figure 4D). These results further supported the hypothesis that PbbZIP TFs may regulate the formation of BS through the GA signaling pathway.

### qRT-PCR analysis of *PbbZIP*s in the exocarp of ‘Huangguan’ pear

The expression levels of *PbbZIP11*, *PbbZIP16*, *PbbZIP53*, *PbbZIP60*, and *PbCPRF2* in ‘Huangguan’ pear with BS were significantly higher than those in normal ‘Huangguan’ pear. After GA_3_ treatment, the expression of the *PbbZIP53*, *PbbZIP60* and *PbHY5* genes in ‘Huangguan’ pears decreased and was significantly lower than that in normal ‘Huangguan’, while the expression of *PbbZIP11* was significantly higher than that in normal ‘Huangguan’ pear (Figure 4E). 

## Discussion

The *bZIP* gene family is a large and complicated family that has many members belonging to different subfamilies. bZIP genes are involved in the responses to abiotic stress and biotic stresses. In a previous study, *bZIP* genes were identified in many species. For example, 50, 116, 47 and 45 *bZIP* genes were identified in *Malus domestica* (apple), *Prunus persica* (peach), *Fragaria vesca* (strawberry) and Chinese jujube, respectively (Wang et al., 2017; Zhang et al., 2020). In the present study, a total of 84 *bZIP* members were identified from white pear, this number is higher than that in *Malus domestica*, *Fragaria vesca* and Chinese jujube and lower than that in *Prunus persica*. These results indicate that *bZIP* gene loss might occur in some genomes.

Phylogenetic analysis showed that the *PbbZIP*s could be divided into 13 subfamilies (Figure 2), which is consistent with the results for *Arabidopsis thaliana*. Notably, some *PbbZIP* genes originally belonging to subgroup I became isolated from their clusters, which has also been seen in *Pyrus communis, Arabidopsis thaliana* and *Vitis vinifera* (Liu et al., 2014). The gene structure and conserved motif results further validated the phylogenetic analysis results (Figure 2). Among the *PbbZIPs*, approximately 23.8% had no introns, and all of them were classified into the S and F groups, which is consistent with previous reports in maize (*Zea mays*) (Wei et al., 2012). In addition, we found that Group D belongs to a specific subgroup, and similar findings have been obtained in celery (*Apium graveolens*).

During evolution, plant genomes have become more complex to adapt to changes in the environment (Fernie and Gutierrez-Marcos, 2019). TFs play important roles in plant environmental adaptation. NAC, bZIP, MYB and WRKY are common TFs in plants that play crucial roles in the regulation of plant hormone-mediated signals for disease and stress resistance. In recent years, increasing evidence has suggested that *bZIP* genes are involved in plant responses to abiotic and biotic stresses, including phytohormone ABA signaling (An et al., 2018), pathogen defense (Thurow et al., 2005; Kaminaka et al., 2006), drought and high salinity (Huang et al., 2016), cold stresses (Shimizu et al., 2005) and light irradiation (Ulm et al., 2004; Osterlund et al., 2000). For example, the *CabZIP1* TF in pepper (*Piper nigrum*) plays a regulatory role in disease defense and stress responses (Lee et al., 2006). In studies of *Arabidopsis thaliana*, transcription of *AtbZIP44* was responsive to temperature, and under ABA, salt and osmotic stress, transcription of *AtbZIP53* was upregulated in seeds, and transcription of *AtbZIP2* was downregulated in roots (Kang et al., 2002; Weltmeier et al., 2009). BS is reported to be associated with light irradiation, sudden drops in temperature, calcium deficiency, and various physiological, developmental and hormonal responses (Li et al., 2008; Wang et al., 2011; Wang et al., 2021). These results provide evidence of the potential involvement of *bZIP* genes in the formation of BS on the surface of ‘Huangguan’ pear.

Functional diversity in *bZIP* genes has been observed in various plant species; for example, *bZIP11* can redundantly repress primary root growth by directly activating IAA3/SHY2 transcription (Weiste et al., 2017), and it participates in regulating the metabolism of trehalose and other minor carbohydrates and amino acid metabolism in *Arabidopsis* by sucrose signaling (Weltmeier et al., 2009; Ma et al., 2011). These observations potentially reflect some valuable functions of its target genes. In the present study, the expression of *PbbZIP11* in ‘Huangguan’ pear with BS was higher than that in CK pear, while the expression of *PbbZIP11* in ‘Huangguan’ pear was decreased significantly after GA_3_ treatment. These results demonstrate that *PbbZIP11* could be induced by GA_3_ and participate in BS formation on the surface of ‘Huangguan’ pear. Our results also provide insights into how bZIPs potentially regulate BS disease through GA signaling. 

## Supplementary material

The following online material is available for this article:

Table S1 - Gene information and primer sequences used for qRT-PCR analysis.

Table S2 - Primary information on bZIP proteins in the genome of pear.

Table S3 - Genomic length, exon number and length of *PbbZIP*s.

Table S4 - Chromosomal locations and syntenic relationships of *PbbZIP* genes.

Table S5 - Detail information and expression of genes involved in BS formation.
