# Supplementary material for: Identification of bZIP transcription factors and their responses to brown spot in pear
Source: Genet Mol Biol. 2022 Jan 31;45(1):e20210175. doi: 10.1590/1678-4685-GMB-2021-0175 (PMC8802300; doi:10.1590/1678-4685-GMB-2021-0175)
Supplement: Table S1 - [file 1415-4757-GMB-45-1-e20210175-s1.pdf]

*Genetics and Molecular Biology*
https://doi.org/10.1590/1678-4685-GMB-2021-0175

# "Supplementary Material to "Identification of bZIP transcription factors and their responses to brown spot in pear"

**Table S1 -** Gene information and primer sequences used for qRT-PCR analysis.

| Gene | Primer sequences | Product length (bp) |
|---|---|---|
| *PbbZIP11* | F:CATGGCTTCTTCAAGTGGGG<br>R:CAGGAGACCAATCTGAGCGG | 189 |
| *PbbZIP16* | F:GGAGACGAAGACAACACCTGC<br>R:CATATGGGTGTGGAGGGGTAC | 202 |
| *PbbZIP17* | F:GGAAATCAACGGAACAACGC<br>R:AGGGAGCACGCACGAGTATG | 191 |
| *PbbZIP44* | F:TGGCCACTGCTGACATGCT<br>R:CACCACCAACCACTAATAACCG | 233 |
| *PbbZIP53* | F:TTGGGATAAATTACCCGAGGA<br>R:GTGTCTGCCGAACCTGATTGT | 222 |
| *PbbZIP60* | F:CCGCCATCATCATCCTCTTC<br>R:GGTCAGTGGAAGGCGAGTTG | 216 |
