# Supplementary material for: Identification of bZIP transcription factors and their responses to brown spot in pear
Source: Genet Mol Biol. 2022 Jan 31;45(1):e20210175. doi: 10.1590/1678-4685-GMB-2021-0175 (PMC8802300; doi:10.1590/1678-4685-GMB-2021-0175)
Supplement: Table S2 - [file 1415-4757-GMB-45-1-e20210175-s2.pdf]

Genetics and Molecular Biology
https://doi.org/10.1590/1678-4685-GMB-2021-0175

"Supplementary Material to "Identification of bZIP transcription factors and their responses to brown spot in pear"

Table S2 - Primary information on bZIP proteins in the genome of pear.

Name	Gene ID	Chromosome	CDS length	Amino acid length	Molecular weight	pI	Predictive subcellular localization
PbBZIP1	Pbr029239.1	Chr1	459	153	37002.51	5.21	Nuclear
PbBZIP2	Pbr021041.1	Chr1	1272	424	103784.48	5.02	Nuclear
PbBZIP3	Pbr012802.1	Chr2	1278	426	106336.11	5.01	Nuclear
PbBZIP4	Pbr015675.1	Chr2	1002	334	81861.27	5.08	Nuclear
PbBZIP5	Pbr022933.1	Chr2	738	246	60257.42	5.14	Nuclear
PbBZIP6	Pbr022894.1	Chr2	2187	729	180005.14	4.92	Nuclear
PbBZIP7	Pbr024746.1	Chr2	909	303	72701.74	5.12	Nuclear
PbBZIP8	Pbr001076.1	Chr2	702	234	59516.09	5.08	Nuclear
PbBZIP9	Pbr040479.1	Chr2	474	158	38717.26	5.22	Nuclear
PbBZIP10	Pbr022685.1	Chr3	429	143	35020.58	5.20	Nuclear
PbBZIP11	Pbr026741.1	Chr3	1062	354	88421.84	5.00	Nuclear
PbBZIP12	Pbr003518.1	Chr3	1542	514	126929.16	4.97	Nuclear
PbBZIP13	Pbr003516.1	Chr3	1263	421	102172.78	5.05	Nuclear
PbBZIP14	Pbr013267.1	Chr3	1362	454	111618.90	5.01	Nuclear
PbBZIP15	Pbr013209.1	Chr3	615	205	51696.62	5.10	Nuclear

Name	Gene ID	Chromosome	CDS length	Amino acid length	Molecular weight	pI	Predictive subcellular localization
PbBZIP16	Pbr013133.1	Chr3	1317	439	109089.12	4.97	Nuclear
PbBZIP17	Pbr013043.1	Chr3	1131	377	91706.00	5.05	Nuclear
PbBZIP18	Pbr010517.1	Chr5	1047	349	87658.63	4.98	Nuclear
PbBZIP19	Pbr030476.1	Chr5	861	287	70264.79	5.06	Nuclear
PbBZIP20	Pbr027414.1	Chr5	774	258	62484.89	5.15	Nuclear
PbBZIP21	Pbr035554.1	Chr5	369	123	30140.67	5.27	Nuclear
PbBZIP22	Pbr025283.1	Chr5	1377	459	113870.28	4.97	Nuclear
PbBZIP23	Pbr014592.1	Chr5	1350	450	110069.20	5.02	Nuclear
PbBZIP24	Pbr014594.1	Chr5	1533	511	126201.19	4.97	Nuclear
PbBZIP25	Pbr020210.1	Chr6	465	155	38571.02	5.18	Nuclear
PbBZIP26	Pbr014120.1	Chr6	957	319	78273.16	5.14	Nuclear
PbBZIP27	Pbr015119.3	Chr6	1002	334	82957.30	5.05	Nuclear
PbBZIP28	Pbr016302.1	Chr6	1284	428	106290.63	5.03	Nuclear
PbBZIP29	Pbr002928.1	Chr7	726	242	60228.35	5.10	Nuclear
PbBZIP30	Pbr002981.1	Chr7	726	242	60228.35	5.10	Nuclear
PbBZIP31	Pbr009654.1	Chr7	2229	743	183693.94	4.91	Nuclear
PbBZIP32	Pbr009693.1	Chr7	510	170	41850.96	5.20	Nuclear
PbBZIP33	Pbr041663.1	Chr7	1002	334	81937.17	5.08	Nuclear
PbBZIP34	Pbr008557.1	Chr8	1086	362	89039.85	5.05	Nuclear
PbBZIP35	Pbr008558.1	Chr8	987	329	80606.59	5.10	Nuclear
PbBZIP36	Pbr018746	Chr8	945	315	77155.28	5.09	Nuclear

Name	Gene ID	Chromosome	CDS length	Amino acid length	Molecular weight	pI	Predictive subcellular localization
	.1						
PbBZIP37	Pbr028080.1	Chr8	1728	576	141366.57	4.95	Nuclear
PbBZIP38	Pbr028081.1	Chr8	1515	505	124372.58	4.98	Nuclear
PbBZIP39	Pbr022222.1	Chr9	609	203	51409.73	5.12	Nuclear
PbBZIP40	Pbr029701.1	Chr9	1044	348	83949.56	5.07	Nuclear
PbBZIP41	Pbr030604.1	Chr9	1341	447	110994.28	4.99	Nuclear
PbBZIP42	Pbr009074.1	Chr10	1047	349	87682.68	5.05	Nuclear
PbBZIP43	Pbr019461.1	Chr10	921	307	75190.30	5.05	Nuclear
PbBZIP44	Pbr020743.1	Chr10	807	269	65307.20	5.14	Nuclear
PbBZIP45	Pbr036339.1	Chr10	1026	342	85837.44	4.98	Nuclear
PbBZIP46	Pbr042765.1	Chr10	807	269	65339.26	5.14	Nuclear
PbBZIP47	Pbr017284.1	Chr11	1050	350	88080.80	5.01	Nuclear
PbBZIP48	Pbr030829.1	Chr11	426	142	34915.64	5.19	Nuclear
PbBZIP49	Pbr036605.1	Chr11	1539	513	127084.01	4.97	Nuclear
PbBZIP50	Pbr038249.1	Chr11	1056	352	88119.40	5.00	Nuclear
PbBZIP51	Pbr004364.1	Chr12	1461	487	120776.18	4.96	Nuclear
PbBZIP52	Pbr017778.1	Chr12	1320	440	105631.20	5.03	Nuclear
PbBZIP53	Pbr028659.1	Chr12	1359	453	111177.22	5.02	Nuclear
PbBZIP54	Pbr035863.1	Chr12	1338	446	110860.90	5.00	Nuclear
PbBZIP55	Pbr018534.1	Chr13	483	161	39890.08	5.19	Nuclear
PbBZIP56	Pbr018536.1	Chr13	429	143	35128.70	5.21	Nuclear

Name	Gene ID	Chromosome	CDS length	Amino acid length	Molecular weight	pI	Predictive subcellular localization
PbBZIP57	Pbr027468.1	Chr13	1572	524	129393.07	4.97	Nuclear
PbBZIP58	Pbr030038.1	Chr13	615	205	51608.22	5.11	Nuclear
PbBZIP59	Pbr034805.1	Chr13	846	282	73106.83	5.03	Nuclear
PbBZIP60	Pbr007163.1	Chr14	639	213	54582.04	5.13	Nuclear
PbBZIP61	Pbr007589.1	Chr14	1326	442	106260.08	5.03	Nuclear
PbBZIP62	Pbr026723.2	Chr14	918	306	72968.14	5.15	Nuclear
PbBZIP63	Pbr002622.1	Chr15	510	170	39988.34	5.22	Nuclear
PbBZIP64	Pbr005860.1	Chr15	1734	578	142187.51	4.95	Nuclear
PbBZIP65	Pbr005861.1	Chr15	1584	528	130031.98	4.97	Nuclear
PbBZIP66	Pbr005914.1	Chr15	474	158	38946.34	5.17	Nuclear
PbBZIP67	Pbr009262.1	Chr15	1047	349	85399.04	5.05	Nuclear
PbBZIP68	Pbr017262.1	Chr15	1050	350	84480.35	5.06	Nuclear
PbBZIP69	Pbr019779.1	Chr15	459	153	36880.16	5.22	Nuclear
PbBZIP70	Pbr026913.1	Chr15	1089	363	89452.94	5.06	Nuclear
PbBZIP71	Pbr027818.1	Chr15	495	165	38527.54	5.23	Nuclear
PbBZIP72	Pbr031203.1	Chr15	1053	351	88965.04	5.03	Nuclear
PbBZIP73	Pbr033760.1	Chr15	603	201	50546.63	5.12	Nuclear
PbBZIP74	Pbr037165.1	Chr16	1230	410	100300.03	5.03	Nuclear
PbBZIP75	Pbr016568.1	Chr17	609	203	51459.80	5.13	Nuclear
PbBZIP76	Pbr017979.1	Chr17	459	153	37002.51	5.21	Nuclear
PbBZIP77	Pbr022503	Chr17	888	296	73866.48	5.05	Nuclear

Name	Gene ID	Chromosome	CDS length	Amino acid length	Molecular weight	pI	Predictive subcellular localization
	.1						
PbBZIP78	Pbr002338.1	scaffold1099.0	1260	420	104000.22	5.04	Nuclear
PbBZIP79	Pbr003750.1	scaffold1170.0	726	242	60094.16	5.11	Nuclear
PbBZIP80	Pbr005556.1	scaffold1282.0	1584	528	129985.89	4.97	Nuclear
PbBZIP81	Pbr005557.1	scaffold1282.0	1734	578	142205.61	4.95	Nuclear
PbBZIP82	Pbr006046.1	scaffold1301.0	669	223	54007.24	5.16	Nuclear
PbBZIP83	Pbr010436.1	scaffold170.2.1	969	323	77427.37	5.09	Nuclear
PbBZIP84	Pbr040390.1	scaffold888.0	1368	456	109558.88	5.05	Nuclear
