# Supplementary material for: Identification of bZIP transcription factors and their responses to brown spot in pear
Source: Genet Mol Biol. 2022 Jan 31;45(1):e20210175. doi: 10.1590/1678-4685-GMB-2021-0175 (PMC8802300; doi:10.1590/1678-4685-GMB-2021-0175)
Supplement: Table S3 - [file 1415-4757-GMB-45-1-e20210175-s3.pdf]

Genetics and Molecular Biology
https://doi.org/10.1590/1678-4685-GMB-2021-0175

“Supplementary Material to “Identification of bZIP transcription factors and their responses to brown spot in pear”

Table S3 - Genomic length, exon number and length of PbbZIPs.

Name	Gene ID	Genomic length(bp)	Exon number	Exon length
PbBZIP1	Pbr029239.1	776	1	776
PbBZIP2	Pbr021041.1	3070	12	1272
PbBZIP3	Pbr012802.1	2422	6	1278
PbBZIP4	Pbr015675.1	7494	8	1563
PbBZIP5	Pbr022933.1	1005	1	1005
PbBZIP6	Pbr022894.1	3636	2	2480
PbBZIP7	Pbr024746.1	6021	2	5934
PbBZIP8	Pbr001076.1	702	1	702
PbBZIP9	Pbr040479.1	1263	1	1263
PbBZIP10	Pbr022685.1	1331	1	1331
PbBZIP11	Pbr026741.1	2330	4	1333
PbBZIP12	Pbr003518.1	7673	12	1542
PbBZIP13	Pbr003516.1	3136	4	1263
PbBZIP14	Pbr013267.1	3205	9	1362
PbBZIP15	Pbr013209.1	615	1	615
PbBZIP16	Pbr013133.1	2663	4	1624
PbBZIP17	Pbr013043.1	4511	5	1131
PbBZIP18	Pbr010517.1	3817	4	1047
PbBZIP19	Pbr030476.1	987	2	861
PbBZIP20	Pbr027414.1	774	1	774
PbBZIP21	Pbr035554.1	643	3	369
PbBZIP22	Pbr025283.1	2891	6	1732
PbBZIP23	Pbr014592.1	3895	5	1350
PbBZIP24	Pbr014594.1	6898	12	1533
PbBZIP25	Pbr020210.1	465	1	465

Name	Gene ID	Genomic length(bp)	Exon number	Exon length
PbBZIP26	Pbr014120.1	2526	6	957
PbBZIP27	Pbr015119.3	1366	2	1002
PbBZIP28	Pbr016302.1	5786	12	1284
PbBZIP29	Pbr002928.1	2293	4	726
PbBZIP30	Pbr002981.1	2293	4	726
PbBZIP31	Pbr009654.1	4282	2	2605
PbBZIP32	Pbr009693.1	510	1	510
PbBZIP33	Pbr041663.1	6918	8	1572
PbBZIP34	Pbr008557.1	2323	8	1086
PbBZIP35	Pbr008558.1	1420	3	987
PbBZIP36	Pbr018746.1	2876	6	945
PbBZIP37	Pbr028080.1	2934	4	1728
PbBZIP38	Pbr028081.1	2605	4	1515
PbBZIP39	Pbr022222.1	742	1	742
PbBZIP40	Pbr029701.1	4209	11	2250
PbBZIP41	Pbr030604.1	6017	10	1341
PbBZIP42	Pbr009074.1	2211	6	1047
PbBZIP43	Pbr019461.1	4748	5	1510
PbBZIP44	Pbr020743.1	2803	2	2236
PbBZIP45	Pbr036339.1	4061	4	1511
PbBZIP46	Pbr042765.1	1665	2	1098
PbBZIP47	Pbr017284.1	2602	5	1050
PbBZIP48	Pbr030829.1	1351	1	1351
PbBZIP49	Pbr036605.1	6655	11	1539
PbBZIP50	Pbr038249.1	2420	4	1471
PbBZIP51	Pbr004364.1	3444	6	1678
PbBZIP52	Pbr017778.1	2686	4	1794
PbBZIP53	Pbr028659.1	5558	11	1359
PbBZIP54	Pbr035863.1	7416	10	1338
PbBZIP55	Pbr018534.1	1401	1	1401
PbBZIP56	Pbr018536.1	429	1	429
PbBZIP57	Pbr027468.1	3252	10	1572
PbBZIP58	Pbr030038.1	894	1	894

Name	Gene ID	Genomic length(bp)	Exon number	Exon length
PbBZIP59	Pbr034805.1	1468	3	846
PbBZIP60	Pbr007163.1	639	1	639
PbBZIP61	Pbr007589.1	2787	4	1853
PbBZIP62	Pbr026723.2	2626	7	918
PbBZIP63	Pbr002622.1	2735	4	1000
PbBZIP64	Pbr005860.1	2944	4	1734
PbBZIP65	Pbr005861.1	2759	4	1584
PbBZIP66	Pbr005914.1	1555	1	1555
PbBZIP67	Pbr009262.1	2876	6	1408
PbBZIP68	Pbr017262.1	2736	11	1050
PbBZIP69	Pbr019779.1	459	1	459
PbBZIP70	Pbr026913.1	2464	8	1089
PbBZIP71	Pbr027818.1	2761	4	1025
PbBZIP72	Pbr031203.1	4038	4	2175
PbBZIP73	Pbr033760.1	778	1	778
PbBZIP74	Pbr037165.1	7623	12	1896
PbBZIP75	Pbr016568.1	808	1	808
PbBZIP76	Pbr017979.1	776	1	776
PbBZIP77	Pbr022503.1	3477	4	1304
PbBZIP78	Pbr002338.1	6818	12	1260
PbBZIP79	Pbr003750.1	2302	4	726
PbBZIP80	Pbr005556.1	2763	4	1584
PbBZIP81	Pbr005557.1	3010	4	1734
PbBZIP82	Pbr006046.1	1764	4	1451
PbBZIP83	Pbr010436.1	4265	3	2289
PbBZIP84	Pbr040390.1	2420	4	1368
