# Supplementary material for: Identification of bZIP transcription factors and their responses to brown spot in pear
Source: Genet Mol Biol. 2022 Jan 31;45(1):e20210175. doi: 10.1590/1678-4685-GMB-2021-0175 (PMC8802300; doi:10.1590/1678-4685-GMB-2021-0175)
Supplement: Table S4 - [file 1415-4757-GMB-45-1-e20210175-s4.pdf]

Genetics and Molecular Biology
https://doi.org/10.1590/1678-4685-GMB-2021-0175

"Supplementary Material to "Identification of bZIP transcription factors and their responses to brown spot in pear"

Table S4 - Chromosomal locations and syntenic relationships of *PbbZIP* genes.

Name	Gene ID	Chromosomal	Starting point	Stoping point	Strand
PbBZIP1	Pbr029239.1	Chr1	2389878	2390653	+
PbBZIP2	Pbr021041.1	Chr1	3250368	3253437	+
PbBZIP3	Pbr012802.1	Chr2	5526442	5528863	-
PbBZIP4	Pbr015675.1	Chr2	6170887	6178380	-
PbBZIP5	Pbr022933.1	Chr2	6976334	6977338	-
PbBZIP6	Pbr022894.1	Chr2	7309361	7312996	-
PbBZIP7	Pbr024746.1	Chr2	8549572	8555592	+
PbBZIP8	Pbr001076.1	Chr2	12231889	12232590	+
PbBZIP9	Pbr040479.1	Chr2	15517927	15519189	+
PbBZIP10	Pbr022685.1	Chr3	1149894	1151224	+
PbBZIP11	Pbr026741.1	Chr3	2967110	2969439	+
PbBZIP12	Pbr003518.1	Chr3	16831076	16838748	+
PbBZIP13	Pbr003516.1	Chr3	16858552	16861687	-
PbBZIP14	Pbr013267.1	Chr3	21256215	21259419	-
PbBZIP15	Pbr013209.1	Chr3	21719032	21719646	-
PbBZIP16	Pbr013133.1	Chr3	22249661	22252323	+
PbBZIP17	Pbr013043.1	Chr3	22992061	22996571	-
PbBZIP18	Pbr010517.1	Chr5	2957830	2961646	-
PbBZIP19	Pbr030476.1	Chr5	2296058	2297044	+
PbBZIP20	Pbr027414.1	Chr5	12967287	12968060	+
PbBZIP21	Pbr035554.1	Chr5	15980857	15981499	-
PbBZIP22	Pbr025283.1	Chr5	19163038	19165928	+
PbBZIP23	Pbr014592.1	Chr5	22847185	22851079	+
PbBZIP24	Pbr014594.1	Chr5	22872574	22879471	-
PbBZIP25	Pbr020210.1	Chr6	4248339	4248803	-
PbBZIP26	Pbr014120.1	Chr6	9323825	9326350	-
PbBZIP27	Pbr015119.3	Chr6	19889864	19891229	+
PbBZIP28	Pbr016302.1	Chr6	21111673	21117458	+
PbBZIP29	Pbr002928.1	Chr7	12429663	12431955	+
PbBZIP30	Pbr002981.1	Chr7	12802639	12804931	-
PbBZIP31	Pbr009654.1	Chr7	1362620	1366901	+

Name	Gene ID	Chromosomal	Starting point	Stoping point	Strand
PbBZIP32	Pbr009693.1	Chr7	1703894	1704403	+
PbBZIP33	Pbr041663.1	Chr7	2182780	2189697	-
PbBZIP34	Pbr008557.1	Chr8	2230491	2232813	+
PbBZIP35	Pbr008558.1	Chr8	2197741	2199160	-
PbBZIP36	Pbr018746.1	Chr8	10553837	10556712	-
PbBZIP37	Pbr028080.1	Chr8	14864215	14867148	-
PbBZIP38	Pbr028081.1	Chr8	14868512	14871116	-
PbBZIP39	Pbr022222.1	Chr9	18881465	18882206	+
PbBZIP40	Pbr029701.1	Chr9	14021920	14026128	+
PbBZIP41	Pbr030604.1	Chr9	19283974	19289990	-
PbBZIP42	Pbr009074.1	Chr10	10127839	10130049	+
PbBZIP43	Pbr019461.1	Chr10	22800599	22805346	-
PbBZIP44	Pbr020743.1	Chr10	17290780	17293582	-
PbBZIP45	Pbr036339.1	Chr10	19091541	19095601	-
PbBZIP46	Pbr042765.1	Chr10	17608035	17609699	+
PbBZIP47	Pbr017284.1	Chr11	24819436	24822037	+
PbBZIP48	Pbr030829.1	Chr11	565147	566497	+
PbBZIP49	Pbr036605.1	Chr11	18152763	18159417	-
PbBZIP50	Pbr038249.1	Chr11	4628100	4630519	+
PbBZIP51	Pbr004364.1	Chr12	2057108	2060551	-
PbBZIP52	Pbr017778.1	Chr12	20360616	20363301	-
PbBZIP53	Pbr028659.1	Chr12	12490432	12495989	+
PbBZIP54	Pbr035863.1	Chr12	16253048	16260463	-
PbBZIP55	Pbr018534.1	Chr13	7268883	7270283	+
PbBZIP56	Pbr018536.1	Chr13	7319005	7319433	+
PbBZIP57	Pbr027468.1	Chr13	2302476	2305727	-
PbBZIP58	Pbr030038.1	Chr13	4056908	4057801	-
PbBZIP59	Pbr034805.1	Chr13	14373472	14374939	+
PbBZIP60	Pbr007163.1	Chr14	14914116	14914754	-
PbBZIP61	Pbr007589.1	Chr14	344099	346885	+
PbBZIP62	Pbr026723.2	Chr14	8763592	8766217	-
PbBZIP63	Pbr002622.1	Chr15	999456	1002190	+
PbBZIP64	Pbr005860.1	Chr15	2664599	2667542	-
PbBZIP65	Pbr005861.1	Chr15	2671458	2674216	-
PbBZIP66	Pbr005914.1	Chr15	3000081	3001635	-
PbBZIP67	Pbr009262.1	Chr15	3989301	3992176	+
PbBZIP68	Pbr017262.1	Chr15	20035048	20037783	+
PbBZIP69	Pbr019779.1	Chr15	6992833	6993291	-
PbBZIP70	Pbr026913.1	Chr15	28456413	28458876	-
PbBZIP71	Pbr027818.1	Chr15	9823393	9826153	+
PbBZIP72	Pbr031203.1	Chr15	39848596	39852633	-
PbBZIP73	Pbr033760.1	Chr15	30168150	30168927	+
PbBZIP74	Pbr037165.1	Chr16	19058863	19066485	-

Name	Gene ID	Chromosomal	Starting point	Stoping point	Strand
PbBZIP75	Pbr016568.1	Chr17	17890291	17891098	+
PbBZIP76	Pbr017979.1	Chr17	19768805	19769580	-
PbBZIP77	Pbr022503.1	Chr17	2569875	2573351	+
PbBZIP78	Pbr002338.1	scaffold1099.0	54879	61696	-
PbBZIP79	Pbr003750.1	scaffold1170.0	35506	37807	-
PbBZIP80	Pbr005556.1	scaffold1282.0	9370	12132	+
PbBZIP81	Pbr005557.1	scaffold1282.0	15287	18296	+
PbBZIP82	Pbr006046.1	scaffold1301.0	48	1811	+
PbBZIP83	Pbr010436.1	scaffold170.2.1	93882	98146	-
PbBZIP84	Pbr040390.1	scaffold888.0	59641	62060	+
