# Supplementary material for: Identification of bZIP transcription factors and their responses to brown spot in pear
Source: Genet Mol Biol. 2022 Jan 31;45(1):e20210175. doi: 10.1590/1678-4685-GMB-2021-0175 (PMC8802300; doi:10.1590/1678-4685-GMB-2021-0175)
Supplement: Table S5 - [file 1415-4757-GMB-45-1-e20210175-s5.pdf]

Genetics and Molecular Biology
https://doi.org/10.1590/1678-4685-GMB-2021-0175

"Supplementary Material to "Identification of bZIP transcription factors and their responses to brown spot in pear"

Table S5 - Detail information and expression of genes involved in BS formation.

GeneID	Pathway Name	Level 1	Level 2	CK_1 FPKM	CK_2 FPKM	CK_3 FPKM	BS_1 FPKM	BS_2 FPKM	BS_3 FPKM	GA_3_1 FPKM	GA_3_2 FPKM	GA_3_3 FPKM	Description	Abbreviation
103931970	ko04075//Plant hormone signal transduction	Environmental Information Processing	Signal transduction	53.14	57.64	71.39	96.21	94.22	96.37	95.75	90.95	89.46	bZIP transcription factor 53-like	PbbZIP53
103957504	ko04075//Plant hormone signal transduction	Environmental Information Processing	Signal transduction	4.24	5.18	5.34	4.94	12.75	9.47	4.41	5.92	4.81	basic leucine zipper 9-like	PbbZIP9
103961780	ko04075//Plant hormone signal transduction	Environmental Information Processing	Signal transduction	76.59	76.53	76.24	94.71	89.46	95.04	72.16	74.78	90.61	bZIP transcription factor 60	PbbZIP60

GeneID	Pathway Name	Level 1	Level 2	CK_1 FPKM	CK_2 FPKM	CK_3 FPKM	BS_1 FPKM	BS_2 FPKM	BS_3 FPKM	GA_3_1 FPKM	GA_3_2 FPKM	GA_3_3 FPKM	Description	Abbreviation
103928540	ko04075//Plant hormone signal transduction	Environmental Information Processing	Signal transduction	568.91	608.32	568.91	303.02	561.8	190.29	614.45	783.93	589.39	bZIP transcription factor 44	PbbZIP44
103933825	ko04075//Plant hormone signal transduction	Environmental Information Processing	Signal transduction	0.08	0.45	0.96	0.37	0.66	3.24	1.64	2.24	1.5	bZIP transcription factor 2-like	PbbZIP2
103936031	ko04075//Plant hormone signal transduction	Environmental Information Processing	Signal transduction	6.75	8.88	9.81	13.15	11.94	14.19	11.9	11.37	12.28	bZIP transcription factor 16-like	PbbZIP16
103937805	ko04075//Plant hormone signal transduction	Environmental Information Processing	Signal transduction	9.21	11.66	13.05	60.5	25.7	43.56	14.02	13.89	13.51	bZIP transcription factor 11-like	PbbZIP11
103945494	ko04075//Plant hormone signal transduction	Environmental Information Processing	Signal transduction	40.59	38.43	39.53	52.73	43.24	45.06	38.68	39.5	42.94	bZIP transcription factor 17-like	PbbZIP17

GeneID	Pathway Name	Level 1	Level 2	CK_1 FPKM	CK_2 FPKM	CK_3 FPKM	BS_1 FPKM	BS_2 FPKM	BS_3 FPKM	GA_3_1 FPKM	GA_3_2 FPKM	GA_3_3 FPKM	Description	Abbreviation
103928345	ko04075//Plant hormone signal transduction	Environmental Information Processing	Signal transduction	0.29	0.17	0.23	2.75	0.45	2.13	0.11	0.36	0.17	gibberellin receptor GID1C-like	GID1C
103959978	ko01110//Biosynthesis of secondary metabolites;ko00943//Isoflavonoid biosynthesis;ko04075//Plant hormone signal transduction	Metabolism;Environmental Information Processing	Biosynthesis of other secondary metabolites; Signal transduction;Global and overview maps	143.36	63.51	109.01	459.79	93.61	244.51	79.55	51.27	110.71	probable carboxylesterase 15	CES15
103962043	ko04075//Plant hormone signal transduction	Environmental Information Processing	Signal transduction	74.44	120.78	93.31	20.58	63.6	34.17	154.68	118.17	140.04	probable carboxylesterase 18	CES18
103931198	ko04075//Plant hormone	Environmental Information Processing	Signal transduction	1.93	2.81	3.63	8.4	4.51	5.28	2.09	2.59	4.45	scarecrow-like protein 21	SCL21

GeneID	Pathway Name	Level 1	Level 2	CK_1 FPKM	CK_2 FPKM	CK_3 FPKM	BS_1 FPKM	BS_2 FPKM	BS_3 FPKM	GA_3_1 FPKM	GA_3_2 FPKM	GA_3_3 FPKM	Description	Abbreviation
103935014	signal transduction ko04075//Plant hormone signal transduction	Environmental Information Processing	Signal transduction	2.07	0.78	1.37	4.26	3.5	4.48	1.8	2.05	1.9	scarecrow-like protein 22	SCL22
103941523	ko04075//Plant hormone signal transduction	Environmental Information Processing	Signal transduction	1.93	3.48	2.36	10.09	4.71	10.75	6.23	6.85	5.75	scarecrow-like protein 4	SCL4
103954288	ko04075//Plant hormone signal transduction	Environmental Information Processing	Signal transduction	1.61	2.5	1.87	3.98	4.15	5.82	1.76	2.02	1.61	scarecrow-like protein 14	SCL14
103960214	ko04075//Plant hormone signal transduction	Environmental Information Processing	Signal transduction	0.15	0.2	0.26	0.09	0.48	1.29	0.34	0.36	0.2	protein SCARECROW 2-like	SCL2
103963184	ko04075//Plant hormone signal transduction	Environmental Information Processing	Signal transduction	0	0.1	0.04	0.55	0.06	0.27	0.11	0.39	0.03	scarecrow-like protein 15	SCL15

GeneID	Pathway Name	Level 1	Level 2	CK_1 FPKM	CK_2 FPKM	CK_3 FPKM	BS_1 FPKM	BS_2 FPKM	BS_3 FPKM	GA_3_1 FPKM	GA_3_2 FPKM	GA_3_3 FPKM	Description	Abbreviation
103964673	ko04075//Plant hormone signal transduction	Environmental Information Processing	Signal transduction	6.64	5.8	6.77	13.08	10.45	15.52	7.35	6.87	8.38	scarecrow-like protein 33	SCL33
103964676	ko04075//Plant hormone signal transduction	Environmental Information Processing	Signal transduction	2.09	0.99	1.42	10.92	9.49	18.32	1.82	1.97	1.84	scarecrow-like protein 30	SCL30
103964678	ko04075//Plant hormone signal transduction	Environmental Information Processing	Signal transduction	30.52	19.6	25.63	65.77	52.21	70.38	26.87	23.76	29.35	scarecrow-like protein 11	SCL11
103927146	ko04075//Plant hormone signal transduction;ko04712//Circadian rhythm - plant	Environmental Information Processing;Organismal Systems	Signal transduction;Environmental adaptation	8.72	3.9	7.11	2.75	3.68	1.1	5.33	3.56	4.19	transcription factor bHLH137-like	bHLH137
103929400	ko04712//Circadian rhythm -	Organismal Systems;Environmental	Environmental adaptation;S	8.14	3.92	3.83	23.12	6.74	16.76	3.09	4.92	2.55	transcription factor bHLH68-	bHLH68

GeneID	Pathway Name	Level 1	Level 2	CK_1 FPKM	CK_2 FPKM	CK_3 FPKM	BS_1 FPKM	BS_2 FPKM	BS_3 FPKM	GA_3_1 FPKM	GA_3_2 FPKM	GA_3_3 FPKM	Description	Abbreviation
	plant;ko04075 //Plant hormone signal transduction	Information Processing	ignal transduction										like isoform X1	
103932057	ko04712//Circadian rhythm - plant;ko04075 //Plant hormone signal transduction	Organismal Systems;Environmental Information Processing	Signal transduction ;Environmental adaptation	13.13	6.18	8.19	29.12	16.82	34.43	9.9	8.14	11.29	transcription factor bHLH128	bHLH128
103936903	ko04712//Circadian rhythm - plant;ko04075 //Plant hormone signal transduction	Environmental Information Processing;Organismal Systems	Environmental adaptation;Signal transduction	2.06	2.26	1.65	0.63	1.68	0.34	2.11	2.08	1.52	transcription factor bHLH113-like	bHLH113

GeneID	Pathway Name	Level 1	Level 2	CK_1 FPKM	CK_2 FPKM	CK_3 FPKM	BS_1 FPKM	BS_2 FPKM	BS_3 FPKM	GA_3_1 FPKM	GA_3_2 FPKM	GA_3_3 FPKM	Description	Abbreviation
103939157	ko04075//Plant hormone signal transduction	Environmental Information Processing	Signal transduction	3.09	1.1	1.96	10.44	10.02	12.3	2.61	2.12	4.06	transcription factor bHLH48-like	bHLH48
103949094	ko04075//Plant hormone signal transduction	Environmental Information Processing	Signal transduction	0.08	0.31	0.04	0.31	0.47	2.09	0.4	0.55	0.24	transcription factor bHLH66-like	bHLH66
103961385	ko03010//Ribosome;ko04075//Plant hormone signal transduction	Environmental Information Processing;Genetic Information Processing	Translation; Signal transduction	0.73	2.04	1.44	6.34	8.44	11.64	2.79	1.98	2.23	transcription factor bHLH62	bHLH62
103962722	ko04075//Plant hormone signal transduction	Environmental Information Processing	Signal transduction	4.7	3.86	3.97	12.37	6.13	7.97	4.23	3.15	5.29	transcription factor bHLH145	bHLH145
103966999	ko04712//Circadian rhythm - plant;ko04075	Organismal Systems;Environmental	Signal transduction ;Environme	3.26	1.43	1.41	0.65	1.46	0	1.85	1.61	1.24	transcription factor bHLH49-	bHLH49

GeneID	Pathway Name	Level 1	Level 2	CK_1 FPKM	CK_2 FPKM	CK_3 FPKM	BS_1 FPKM	BS_2 FPKM	BS_3 FPKM	GA_3_1 FPKM	GA_3_2 FPKM	GA_3_3 FPKM	Description	Abbreviation
	//Plant hormone signal transduction	Information Processing	ntal adaptation										like isoform X2	

	GeneID	Pathway Name	Level 1	Level 2	CK_1 FPKM	CK_2 FPKM	CK_3 FPKM	BS_1 FPKM	BS_2 FPKM	BS_3 FPKM	GA_3_1 FPKM	GA_3_2 FPKM	GA_3_3 FPKM	Description	Abbreviation
**bZIP family TFs**	103931970	ko04075//Plant hormone signal transduction	Environmental Information Processing	Signal transduction	53.14	57.64	71.39	96.21	94.22	96.37	95.75	90.95	89.46	bZIP transcription factor 53-like	PbbZIP53
	103957504	ko04075//Plant hormone signal transduction	Environmental Information Processing	Signal transduction	4.24	5.18	5.34	4.94	12.75	9.47	4.41	5.92	4.81	basic leucine zipper 9-like	PbbZIP9
	103961780	ko04075//Plant hormone signal transduction	Environmental Information Processing	Signal transduction	76.59	76.53	76.24	94.71	89.46	95.04	72.16	74.78	90.61	bZIP transcription factor 60	PbbZIP60
	103928540	ko04075//Plant hormone signal transduction	Environmental Information Processing	Signal transduction	568.91	608.32	568.91	303.02	561.8	190.29	614.45	783.93	589.39	bZIP transcription factor 44	PbbZIP44
	103933825	ko04075//Plant hormone signal transduction	Environmental Information Processing	Signal transduction	0.08	0.45	0.96	0.37	0.66	3.24	1.64	2.24	1.5	bZIP transcription factor 2-like	PbbZIP2
	103936031	ko04075//Plant hormone signal transduction	Environmental Information Processing	Signal transduction	6.75	8.88	9.81	13.15	11.94	14.19	11.9	11.37	12.28	bZIP transcription factor 16-like	PbbZIP16
	103937805	ko04075//Plant hormone signal transduction	Environmental Information Processing	Signal transduction	9.21	11.66	13.05	60.5	25.7	43.56	14.02	13.89	13.51	bZIP transcription factor 11-like	PbbZIP11

	103945494	ko04075//Plant hormone signal transduction	Environmental Information Processing	Signal transduction	40.59	38.43	39.53	52.73	43.24	45.06	38.68	39.5	42.94	bZIP transcription factor 17-like	PbbZIP17
Gibberellin signal	103928345	ko04075//Plant hormone signal transduction	Environmental Information Processing	Signal transduction	0.29	0.17	0.23	2.75	0.45	2.13	0.11	0.36	0.17	gibberellin receptor GID1C-like	GID1C
	103959978	ko01110//Biosynthesis of secondary metabolites;ko00943//Isoflavonoid biosynthesis;ko04075//Plant hormone signal transduction	Metabolism;Environmental Information Processing	Biosynthesis of other secondary metabolites;Signal transduction;Global and overview maps	143.36	63.51	109.01	459.79	93.61	244.51	79.55	51.27	110.71	probable carboxylesterase 15	CES15
	103962043	ko04075//Plant hormone signal transduction	Environmental Information Processing	Signal transduction	74.44	120.78	93.31	20.58	63.6	34.17	154.68	118.17	140.04	probable carboxylesterase 18	CES18
	103931198	ko04075//Plant hormone	Environmental Information Processing	Signal transduction	1.93	2.81	3.63	8.4	4.51	5.28	2.09	2.59	4.45	scarecrow-like protein 21	SCL21

	signal transduction													
103935014	ko04075//Plant hormone signal transduction	Environmental Information Processing	Signal transduction	2.07	0.78	1.37	4.26	3.5	4.48	1.8	2.05	1.9	scarecrow-like protein 22	SCL22
103941523	ko04075//Plant hormone signal transduction	Environmental Information Processing	Signal transduction	1.93	3.48	2.36	10.09	4.71	10.75	6.23	6.85	5.75	scarecrow-like protein 4	SCL4
103954288	ko04075//Plant hormone signal transduction	Environmental Information Processing	Signal transduction	1.61	2.5	1.87	3.98	4.15	5.82	1.76	2.02	1.61	scarecrow-like protein 14	SCL14
103960214	ko04075//Plant hormone signal transduction	Environmental Information Processing	Signal transduction	0.15	0.2	0.26	0.09	0.48	1.29	0.34	0.36	0.2	protein SCARECROW 2-like	SCL2
103963184	ko04075//Plant hormone signal transduction	Environmental Information Processing	Signal transduction	0	0.1	0.04	0.55	0.06	0.27	0.11	0.39	0.03	scarecrow-like protein 15	SCL15
103964673	ko04075//Plant hormone signal transduction	Environmental Information Processing	Signal transduction	6.64	5.8	6.77	13.08	10.45	15.52	7.35	6.87	8.38	scarecrow-like protein 33	SCL33

103964676	ko04075//Plant hormone signal transduction	Environmental Information Processing	Signal transduction	2.09	0.99	1.42	10.92	9.49	18.32	1.82	1.97	1.84	scarecrow-like protein 30	SCL30
103964678	ko04075//Plant hormone signal transduction	Environmental Information Processing	Signal transduction	30.52	19.6	25.63	65.77	52.21	70.38	26.87	23.76	29.35	scarecrow-like protein 11	SCL11
103927146	ko04075//Plant hormone signal transduction;ko04712//Circadian rhythm - plant	Environmental Information Processing;Organismal Systems	Signal transduction;Environmental adaptation	8.72	3.9	7.11	2.75	3.68	1.1	5.33	3.56	4.19	transcription factor bHLH137-like	bHLH137
103929400	ko04712//Circadian rhythm - plant;ko04075//Plant hormone signal transduction	Organismal Systems;Environmental Information Processing	Environmental adaptation;Signal transduction	8.14	3.92	3.83	23.12	6.74	16.76	3.09	4.92	2.55	transcription factor bHLH68-like isoform X1	bHLH68
103932057	ko04712//Circadian rhythm - plant;ko04075//Plant	Organismal Systems;Environmental	Signal transduction;Environme	13.13	6.18	8.19	29.12	16.82	34.43	9.9	8.14	11.29	transcription factor bHLH128	bHLH128

	hormone signal transduction	Information Processing	ntal adaptation											
103936903	ko04712//Circadian rhythm - plant;ko04075//Plant hormone signal transduction	Environmental Information Processing;Organismal Systems	Environmental adaptation;Signal transduction	2.06	2.26	1.65	0.63	1.68	0.34	2.11	2.08	1.52	transcription factor bHLH113-like	bHLH113
103939157	ko04075//Plant hormone signal transduction	Environmental Information Processing	Signal transduction	3.09	1.1	1.96	10.44	10.02	12.3	2.61	2.12	4.06	transcription factor bHLH48-like	bHLH48
103949094	ko04075//Plant hormone signal transduction	Environmental Information Processing	Signal transduction	0.08	0.31	0.04	0.31	0.47	2.09	0.4	0.55	0.24	transcription factor bHLH66-like	bHLH66
103961385	ko03010//Ribosome;ko04075//Plant hormone signal transduction	Environmental Information Processing;Genetic Information Processing	Translation; Signal transduction	0.73	2.04	1.44	6.34	8.44	11.64	2.79	1.98	2.23	transcription factor bHLH62	bHLH62

103962722	ko04075//Plant hormone signal transduction	Environmental Information Processing	Signal transduction	4.7	3.86	3.97	12.37	6.13	7.97	4.23	3.15	5.29	transcription factor bHLH145	bHLH145
103966999	ko04712//Circadian rhythm - plant;ko04075//Plant hormone signal transduction	Organismal Systems;Environmental Information Processing	Signal transduction;Environmental adaptation	3.26	1.43	1.41	0.65	1.46	0	1.85	1.61	1.24	transcription factor bHLH49-like isoform X2	bHLH49
